# Soybean Synergies: A Comprehensive Review on Novel Extraction Techniques and Their Role in Unlocking Health Potential

**DOI:** 10.1002/fsn3.70983

**Published:** 2025-09-30

**Authors:** Muhammad Rashid, Iqra Khalid, Gaurav Sanghvi, Amar Shankar, Farhan Saeed, Muhammad Afzaal, Mehwish, Mayank Kundlas, Catherine Tamale Ndagire

**Affiliations:** ^1^ Department of Food Science Government College University Faisalabad Faisalabad Pakistan; ^2^ Institute of Biological Sciences Gomal University Dera Ismail Khan Pakistan; ^3^ Department of Microbiology, Faculty of Science, Marwadi University Research Center Marwadi University Rajkot Gujarat India; ^4^ Department of Food Technology, School of Engineering and Technology JAIN (Deemed to Be University) Bangalore Karnataka India; ^5^ Institute of Engineering and Technology, Centre for Research Impact & Outcome Chitkara University Rajpura Punjab India; ^6^ Department of Food Innovation and Nutrition Mountains of the Moon University Fort Portal Uganda

**Keywords:** health benefits, methods of oil extraction, soybean, soybean oil bodies

## Abstract

Food is essential for human nutrition, health, and the development of the human body. Soybean is an important plant‐based food and is considered the primary dietary source in recent days. Almost 18%–22% of soybean is comprised of tiny, stable oil bodies with a concentration of triacylglycerol, rich in bioactive substances. These oil bodies of soybean consist of oleosins and steroleosin as part of the monolayer of phospholipids. When soybean oil bodies (SOBs) are extracted in an aqueous solution, a second layer of proteins, primarily composed of lipoxygenase, glycinin, and conglycinin, including Bd 30 K/P34, is added. Research has been conducted on SOBs to better understand their characteristics and how they interact with other chemicals in the manufacturing of many food products to replace the traditional oil‐in‐wateremulsions method. This review briefly explains the composition of soybean and different oil extraction techniques, as well as its health benefits. Among the plant‐based proteins, soy protein and its byproducts are discussed in detail. Additionally, the review explores the qualities of soybean oil bodies, highlighting their most common and effective food applications and providing a detailed description of their structure and composition.

## Graphical abstract

Almost 18%–22% of soybean is comprised of tiny, stable oil bodies with a concentration of triacylglycerol, rich in bioactive substances. These oil bodies of soybean consist of oleosins, oleosins, and steroleosin as part of the monolayer of phospholipids. When soybean oil bodies (SOBs) are extracted in an aqueous solution, a second layer of proteins, primarily composed of lipoxygenase, glycinin, and conglycinin, including Bd 30 K/P34, is added.

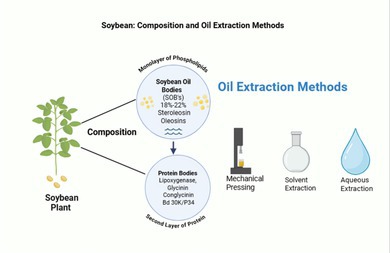

## 1 Introduction

A crop in the legume family is the soybean ( *Glycine max* (L.) Merr.) (Mangena 2023). As a protein‐rich resource and an oil crop, soybeans hold a prominent place in the world economy (Engelbrecht et al. 2020). Soybeans are a significant source of high‐quality protein, lipids, dietary fiber, vitamins, and minerals (Asif and Acharya 2013). It is therefore largely acknowledged as an essential plant‐based source of protein with broad consequences for nutrition and the health of humans and animals. The advent of vegetarianism and the current boom in health‐conscious eating habits have highlighted the growing demand from consumers for plant‐based protein sources (Pointke et al. 2022). The soybean, which is praised for its abundance of high‐quality protein, has been especially highlighted by this trend (Corgneau et al. 2019). It is grown on more than 137.10 million hectares globally in 2023–2024, with Brazil having the biggest area at 45.8 million hectares. Of those, 153 million tonnes of seeds were harvested, yielding 3.3 t per hectare (United States Department of Agriculture 2024a, 2024b). The USA ranks in second place with 33.3 million hectares of soybean cultivation, yielding 113.27 million tonnes of seeds and 3.4 t per hectare, respectively (United States Department of Agriculture 2024a, 2024b). Then comes Argentina (48.10 million metric tons), Paraguay (48.10 million metric tons), China (20.84 million metric tons), India (11.88 million metric tons), Canada (6.98 million metric tons), and Russia (6.8 million metric tons) (Popescu 2024). Brazil, the USA, Argentina, and Paraguay together account for 91.7% of global soybean production (as presented in Tables 1 and 2). Since soybeans are rich in folic acid and isoflavonoids, they are employed in diets all over the world. Since soybeans and their derivatives contain a large number of necessary amino acids and offer several health benefits, they are regarded as significant plant protein sources. Quality lipids and polyunsaturated fatty acid content in soybeans are also crucial from a nutraceutical standpoint (Tidke et al. 2015).

**TABLE 1:** Per 100 g dry weight, soybean raw greens contain the following nutrients (“USDA Nutrient Data Laboratory.” As of August 10 2016, this article was retrieved).

Water content (%)	68	Saturated fatty acids (g)	2.47
Energy (KJ)	1992	Sugar (g)	0.0
Protein (g)	40.6	Magnesium (mg)	203
Fat (g)	21.4	Phosphorus (mg)	606
Carbohydrates (g)	34	Calcium (mg)	6.16
Fiber (g)	13.1	Iron (mg)	11.09
Poly unsaturated fatty acid (g)	10	Vitamin C (mg)	90.6
Mono unsaturated fatty acids (g)	4	Vitamin A (IU) (mg)	563

**TABLE 2:** Production of soybean in top 8 countries in 2023/2024 (United States Department of Agriculture 2024a, 2024b).

Location	Production, MMT = Million metric tons
Brazil	153
United State of America	113.27
Argentina	48.10
Paraguay	48.10
China	20.84
India	11.88
Canada	6.98
Russia	6.8
Total	408.97

The crop is also known as the miracle bean, golden bean, super legume, or the crop of the planet because of its varied applications and immense significance. All of the essential amino acids that the human body is unable to synthesize can be found in soybeans (Nandakishor et al. 2017). Soybeans, like other legume crops, can fix up to 65–100 kg/ha of atmospheric nitrogen into the soil, increasing soil fertility (Youseif et al. 2014). Due to soybean's nitrogen fixation capability, utilizing soybean seeds for crop rotation is also an important tool in sustainable agriculture. Both human food and animal feed can benefit from soybean seeds' protein and oil content of around 3144% and 1926%, respectively.

One of the most commercially significant leguminous seed crops at the moment is soybean. The need for soybeans is steadily rising in tandem with population growth and societal progress. According to statistical data, between 1961 and 2017, the world's soybean production increased by about 13 times (Ray et al. 2013). A larger planting area is the primary contributor to this production increase, even though breeding efforts also play a role (Rincker et al. 2014).

**FIGURE 1:**
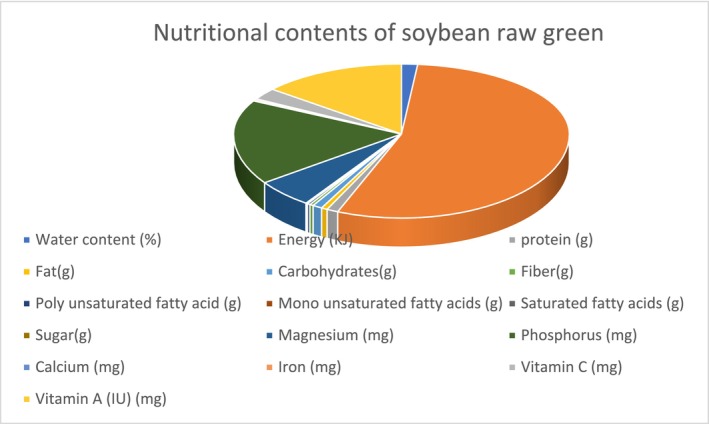
Nutritional contents of raw green soybean.

Soybeans are, furthermore, rich in vitamins and minerals, making them a valuable source of nutrition for people and animals. In addition, soybean crop residues can be used for biofuel production, an important renewable energy source (Barthet and Puvirajah 2021). Additionally, the review explores the qualities of soybean oil bodies, highlighting their most common and effective food applications and providing a detailed description of their structure and composition (Figure 1).

The soybean crop can be used to make a variety of foods and eaten at various phases of development, such as sprouting, vegetable, and mature (Ebert et al. 2017). The well‐known mature soybean is harvested dry and used to make soy‐based products, including natto, soy sauce, tofu, and soybean milk. However, when the seed occupies 80%–90% of the pod cavity, vegetarian soybeans—known as “Maodou” in China and “Edamame” in Japan—are harvested in the immature stage (R6–R7) (Wang et al. 2017). Maodou is a noteworthy source of plant‐based proteins, according to studies.

Food goods like soybean oil and tofu/bean curd, as well as food additives like soy lecithin and industrial additives like colors, plastics, and cosmetics, are all made from soybeans (Modgil et al. 2020). Soybean grain and its derivatives, including soy milk, soy sauce, miso, tofu, and tempeh, have long played a significant role in Asian diets. On the basis of this, pastes and spreads are created as alternatives to dairy products (soy milk, cheese), meat substitutes, and cereal items (bread, pasta, flour) (Wilk 2017). There are a lot of soy products on the market right now, and demand for them is only increasing.

**FIGURE 2:**
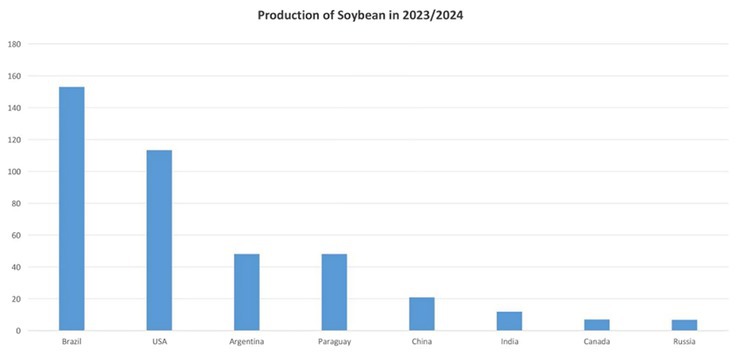
Graphical presentation of production of soybean in different regions in 2023–2024.

Vitamins, minerals, fiber, and extremely nutritious protein may all be found in soybeans. The fat from soybeans also contains essential unsaturated fatty acids (Chen et al. 2012). First and foremost, the soybean proteins are globulins, which make up around 70% of all proteins and provide auxiliary purposes. A class of proteins known as albumins includes other proteins with structural and enzymatic roles. Most of them are regulators of proteolytic activity and can generate inactive complexes that affect the biological value and technological use of raw materials used in food production. Approximately 6% of soluble soybean proteins are protease inhibitors (Modgil et al. 2020) (Figure 2).

## 2 Etymology

The Japanese word “soi,” which means “soy sauce” and is a regional variation of “shōyu,” is where the word “soy” originates. Linnaeus is the source of the genus' name, *Glycine*. One of the species that was once in the genus but has since been moved to the genus *Apios* had a delicious root, which Linnaeus noted when naming the genus. “Glykós,” the Greek word for “sweet,” was Latinized based on the sweetness. Globally, soybeans ( *Glycine max* (L.) Merr.) are a significant crop for oil and dietary protein. *G. soja* , the sympatric wild annual progenitor of soybean that is found throughout East Asia, including most of China, Korea, Japan, and a portion of Russia, was domesticated approximately 5000 years ago (Larson et al. 2014). *G. soja* has a lot of potential to improve its agriculturally significant domesticated relative beyond what is now understood because of its higher level of genetic diversity and ability to adapt to severe settings (Zhang et al. 2017; Qi et al. 2014). The majority of *G. soja* research has focused on understanding the history of soybean domestication; relatively little work has been done to exploit it as a genetic reservoir for improving soybeans (Zhang et al. 2016). According to reports, *G. soja* was domesticated between 6000 and 9000 years ago in areas of Central China along the Huang‐Huai valley or Yellow River (Han et al. 2015), giving rise to landraces of *G. max* and, after additional selection, the current (elite) cultivated material. The history of *G. soja* in comparison to *G. max* , however, is far more complicated and involves a number of conflicting theories. The domestication process most likely took place over a long period of time, which allowed for frequent introgressions between farmed and wild populations during that time (Wang and Li 2013). No indication of numerous domestication episodes in East Asia was found, despite the fact that opposing research of candidate domestication locations in Korean wild soybeans supports a single selective sweep (Chung et al. 2013).

Three main hypotheses—the single origin theory, the multiple origin hypothesis, and the complex hypothesis—were recently offered in a study of the history of domestication, which summarized the conflicting theories regarding the genesis and domestication of soybeans (Sedivy et al. 2017). According to the single origin hypothesis, *G. max* and *G. soja* diverged during a single domestication event in Central China no more than 9000 years ago. This theory is supported by the finding that all of the chosen domesticated soybeans clustered together when whole‐genome single nucleotide polymorphism (SNPs) of 302 wild, landrace, and cultivated soybeans were analyzed (Zhou et al. 2015). According to the multiple origin hypothesis, between 5000 and 9000 years ago, *G. max* was domesticated from *G. soja* in a series of occurrences. With a *G. soja* / *G. max* complex initially diverging prior to repeated domestication processes, the complex hypothesis integrates the findings of two recent investigations (Li et al. 2014). Based on a whole‐genome comparison of one wild soybean ecotype to one soybean cultivar, the *G. soja* / *G. max* complex is estimated to have existed 0.27 million years ago (MYA), or 0.8 MYA based on a pan‐genome comparison of seven wild soybean ecotypes.

According to this final theory, domestication would have resulted from a *G. soja* / *G. max* complex that was already divergent (Sedivy et al. 2017). Based on their geographic origins, individuals from the soja or max subpopulations were closely packed (Zhang et al. 2015). One possibility might be that the early‐domesticated *G. soja* or *G. soja* / *G. max* complex traveled from China to Korea and Japan before being domesticated to various degrees to suit local requirements. However, it is generally acknowledged that *G. max* was produced from *G. soja* or the *G. soja* / *G. max* complex by numerous distinct efforts through a protracted, laborious, and intricate domestication process (Sedivy et al. 2017).

## 3 Classification

Wild annuals and farmed soybeans are classified according to the following taxonomy:

- Put in order: Fabales
- Family Fabaceae (Leguminosae)
- A subfamily Papilionoideae
- Phaseoleae tribe
- Glycininae Subtribe
- The genus *Glycine* Willd. Soja Subgenus (Moench) Herm, F.J. Species Soja glycine Species of Zucc and Sieb *Glycine max* Merr (L.) (Singh 2017).

In relation to quality features including nutritional value, stress tolerance, and yield, different soybean varieties differ in their genetic genes, physical purity, and germination capacity (McCarville et al. 2017). These soybean quality characteristics are now the most important factors for producers. The identification of soybean varieties has become an important research topic since various varieties have distinct qualitative qualities. Chemometric technology and genetic analysis are regarded as effective methods for precisely identifying seed kinds (Satturu et al. 2018). Only laboratory settings can use these techniques, and operators must possess professional abilities. Furthermore, these techniques are very ineffective, expensive, and damaging. Furthermore, staff members are susceptible to visual fatigue, and manual detection is quite subjective, especially when there are a lot of samples, and they seem helpless. Thus, a technique that can swiftly and nondestructively detect soybean seeds is desperately needed.

Researchers have been using machine vision, near‐infrared (NIR) spectroscopy, hyperspectral imaging (HSI), and nondestructive determination technologies to identify grains in recent years due to their rapid development. Different types of soybeans exhibit variations in their internal chemical makeup, external appearance (such as texture, color, damage, etc.), and other features. However, it is challenging for machine vision technology to produce appropriate results for soybean varieties with very comparable outward traits. When choosing soybean seed varieties, variations in the chemical makeup of soybeans are just as important as their exterior characteristics. A common method for identifying seed kinds is NIR spectroscopy. In order to do the corresponding analysis, the corresponding functional groups or corresponding chemical compounds can be identified using spectral fingerprint analysis of the high vibration frequency absorption of chemical bonds like C‐H, O‐H, and N‐H in the NIR range between 780 and 2500 nm (Zhang and Ji 2019). Nevertheless, the NIR spectroscopy method is unsatisfactory in terms of detection accuracy because it can only gather spectral information in a portion of the sample.

By combining spectrum analysis and machine vision technologies, HSI technology is able to extract the sample's spectral and picture information (Sun et al. 2020). Hyperspectral technology is unique in the identification and quality inspection of agricultural products because it can obtain hyperspectral cube data of many samples at once, overcoming the limitations of traditional spectral analysis and machine vision technologies. Chemical composition prediction (Zhang and Guo 2019), variety (Cao et al. 2018; Zhu et al. 2019) and quality identification (Liu, Li, et al. 2020; Liu, Zhang, et al. 2020) have made extensive use of HSI technology.

An accurate, rapid, and reliable method for detecting soybean varieties is extremely important during processing and planting.

## 4 Stages of Soybean Growth

**FIGURE 3:**
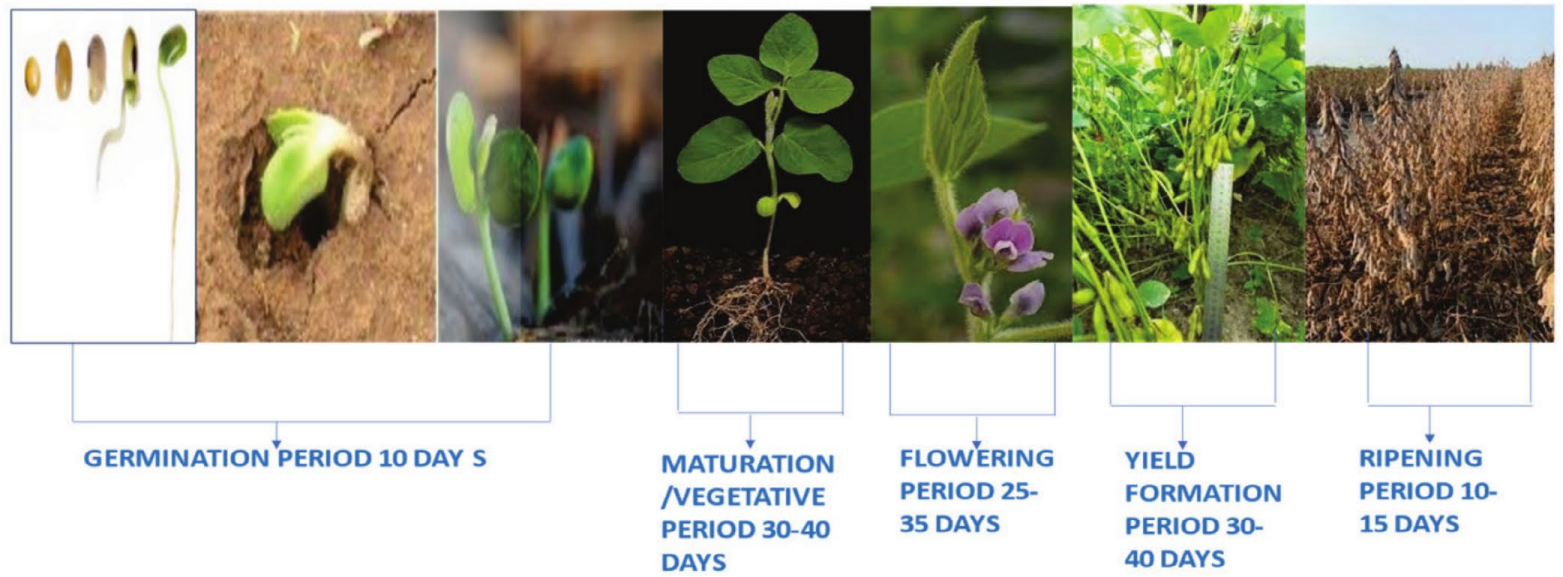
Stages of soybean growth.

Soybeans often grow in two ways: determinate and indeterminate. In the soybean plant, the vegetative and reproductive phases make up the biological cycle. The seed absorbs water to initiate the germination process, which marks the beginning of the vegetative phase. The emergence of the first flower buds in soybean varieties with a determinate growth pattern, or the first raceme in varieties with an indeterminate development behavior, marks the end of the vegetative phase and the start of the reproductive stage. At harvest, the reproductive phase comes to a close (Lenssen and Wright 2013) (Figure 3).

### 4.1 Vegetative Stages

See Table 3 for details.

**TABLE 3:** Vegetative phase of soybean growth (Singh 2017).

Stages of vegetative growth	Description
VE (Emergence)	The earth surface has been penetrated by cotyledons
VC (Cotyledon)	Expanding unifoliolate leaves with a single node
V1 (first trifoliolate)	Two nodes and a single set of fully unfurled trifoliolate leaves
V2 (second trifoliolate)	Three nodes and two pairs of trifoliolate leaves unfolded
V4 (four trifoliolate)	There are four unfolded trifoliolate leaves
V(*n*) (*n*th trifoliolate)	Trifoliolate leaves continue to unfold in the V stages; the final number of trifoliolate depends on the soybean variety and the surrounding circumstances

### 4.2 Reproductive Stages

See Table 4 for details.

**TABLE 4:** Reproductive phases of soybean growth (Singh 2017).

R1	At least one blossom is visible on the main stem and is starting to bloom
R2	Flowers on either of the top two nodes are in full bloom
R3	Pods on one of the top four nodes are 4.8 mm long at the start of the pod set
R4	On one of the top four nodes, the entire pod measures 19 mm in length
R5	Starting seeds on one of the top four nodes measure 3.2 mm in length
R6	Complete seeds: seeds on one of the top four nodes fill the pods completely
R7	One ripe pod turned up on the plant at the onset of maturity
R8	95% of the pods have attained full maturity and developed their mature color

#### 4.2.1 Soybean Maturity Class

The length of each developmental stage depends on the maturity class. While early‐maturing soybean varieties may grow fewer leaves and move through the developmental phases more quickly, late‐maturing soybean varieties may develop more leaves and move through the stages more slowly (SDSU Extension 2019).

#### 4.2.2 Photoperiod

Daylength and photoperiod have an impact on soybean plants, and the plants' reaction to these factors controls when they blossom. The way different types of soybeans react to photoperiod varies. While some types need longer days to begin flowering, others will do so in comparatively shorter amounts of time. When compared to other soybean varieties, those acclimated to the northern U.S. begin flowering under longer days (South Dakota's summer days last about 14 h) (SDSU Extension 2019).

#### 4.2.3 Soybean Seed Resilience

Unpredictable rainfall patterns brought on by global climate change have consistently threatened soybean output, particularly in rainfed areas (Luan et al. 2021). Drought is a complicated stressor that impacts different morpho‐physiological characteristics at every stage of growth and causes significant financial losses (Wei et al. 2018). Water needs for soybeans are minimal during the vegetative stage, peak during the flowering‐to‐pod‐filling period, and then decrease as the plant ages (Lamichhane et al. 2020). During the vegetative stages, mild drought stress can lower soybean growth (plant height) and grain production; however, if the stress is terminated at this point, soybeans may be able to make up for the water stress (Wang et al. 2022). On the other hand, any level of dryness throughout the stage of flowering to pod setting can have a substantial and permanent detrimental impact on soybean output and growth (Pinnamaneni et al. 2021).

The primary source of drought‐induced damage to soybeans at any stage of growth is the suppression and disturbance of photosynthesis, which is essential for sustaining plant growth and recovery following drought (Iqbal et al. 2019). For decades, researchers have used photosynthetic‐related characteristics, especially chlorophyll concentration, to screen genotypes for drought tolerance (Sakoda et al. 2021). These research results demonstrated that cultivars that can withstand drought have larger levels of chlorophyll or can at least sustain them, which could lead to increased photosynthetic rate and production (Guzzo et al. 2020).

### 4.3 Nitrogen‐Fixing Ability

The production and quality of soybeans depend on soil, weather, and agrotechnical conditions, including mineral fertilization and inoculation of seeds. The primary elements influencing the yield and chemical makeup of soybean seeds are nitrogen fertilization and seed inoculation. Because of their symbiotic relationship with the bacteria *Bradyrhizobium japonicum* , soybeans are able to fix free atmospheric nitrogen. Because of this phenomenon, soybeans can provide up to 100 kg/ha of nitrogen to the soil, which lowers the need for nitrogen fertilizer (Griebsch et al. 2020). Due to its high nitrogen (N) requirements, soybeans must absorb 80 kg of N for every Mg of seed produced. Symbiotic soil bacteria use biological nitrogen fixation (BNF) to supply 50%–60% and even 90% of nitrogen on average (Ciampitti and Salvagiotti 2018).

The growth, development, and yield of soybeans are impacted by the conversion of atmospheric nitrogen into the form of ammonium that plants can use. Biological N fixing needs to be carried out as efficiently as possible in order to produce a high seed yield of soybeans and meet their nitrogen (N) needs (Moretti et al. 2018). Since Central European soils do not contain *B. japonicum* , soybean seeds are injected with these bacteria before being sown (Narożna et al. 2015). In Europe, Australia, and America, inoculation of soybeans is a frequent and ubiquitous method that has been shown to increase the species' productivity (Szpunar‐Krok et al. 2023). Compared to the United States (USA), South America uses this management technique far more frequently.

The practice of seed inoculation is economical and cost‐effective. There are several inoculation techniques available. Before being sown, soybean seeds are often coated with symbiotic bacteria to initiate the process of inoculation. The kind of rhizobia, crop cultivation, and environmental factors are the main determinants of Rhizobium inoculation success (Zimmer et al. 2016). As a result, treating seeds with the right bacteria is popular, which lowers the need for fertilization with mineral nitrogen. Between 30% and 60% of soybeans' nitrogen needs can be met by symbiosis (Księżak and Bojarszczuk 2022).

## 5 Chemical Composition

### 5.1 Nutrition

In addition to being low in saturated fats, soy foods are excellent providers of vitamins, minerals, proteins, and fiber. Textured vegetable protein (TVP), textured soy protein (TSP), miso, soy cheese, tempeh, soy sauce, tamari, roasted or boiled soybeans, soymilk, soy mayonnaise, and tofu are only a few of the many types of soy products that have been made (Jayachandran and Xu 2019). Soy seeds offer significantly larger amounts of minerals (5%) than grain seeds (1%) and include sufficient amounts of elements like calcium, iron, and zinc, whose intake is minimal (Kahraman and Kahraman 2016).

Soy is a nearly complete functional food since it contains isoflavones, soy protein, unsaturated fatty acids, vitamin B complex, fiber, iron, calcium, zinc, and other bioactive substances. From a nutraceutical standpoint, soybeans' levels of polyunsaturated fatty acids and high‐quality fats are particularly crucial (Jayachandran and Xu 2019). Pectic polysaccharides are a form of vegetable fiber that makes up the majority of soy fiber. Other peptides, including Bowman‐Birk (a 71 amino acid peptide) and lunasin (a 43 amino acid peptide), are protease inhibitors that have an adverse effect on protein digestion but also have chemopreventive effects in vitro. It has been suggested to use soybean oligosaccharides as sugar substitutes or prebiotics.

Phytic acid (1.0%–2.2%), sterols (0.23%–0.46%), and saponins (0.17%–6.16%) are among the many phytochemicals found in soybeans that have a variety of possible health benefits. Since soybeans are rich in folic acid and isoflavonoids, they are employed in diets all over the world. Due to their high content of essential amino acids and several health benefits, soybeans and their products are regarded as significant plant protein sources (Kamshybayeva et al. 2017).

### 5.2 Proteins

Various healthy diets are formulated using proteins, which are composed of polypeptides that are important for human wellness at any age. A dietary protein provides the body with nitrogen and amino acids, which make up body tissue and make physiological enzymes essential to the body's health. The Protein Digestibility Corrected Amino Acid Score (PDCAAS) assesses the quality of proteins by calculating the essential amino acid content and the actual digestibility of the feces (Hertzler et al. 2020).

The name of the type of protein refers to the type of protein found in plants, like nuts, beans, and whole grains that make up the bulk of the diet. Beans (legumes) provide protein to the human body and are historically found in Asia. Since soy protein has numerous advantages, it has become popular in occidental countries, particularly America, with multiple stocks on the supermarket shelves made with soy protein. Along with rich protein content and bodybuilding, vegan diets seek to replace animal meat with soy‐based protein products (Rizzo and Baroni 2018; Sui et al. 2021).

Soybeans were obtained from the United States, Argentina, South America, China, and India. These are five leading soybean‐producing countries. Soybeans imported from the United States are converted into soybean meals. The soybean meal produced by Argentina contained the least crude protein compared to soybeans grown in China.

Although recent studies reveal varying results, the average protein content of soybeans is thought to be 36.5%. Using dry matter as a basis, the Iowa Soybean Association, for instance, reported an average protein concentration of 37.7% (with a range of 30.9%–42.4%) (Iowa Soybean Association 2025). With an average protein level of 33%, Nebraska is the fourth‐largest producer of soybeans in the United States (Dalla Betta et al. 2023). The “Ultra‐High Protein” soybean, created lately by the food technology company Benson Hill, can produce soy flour with 60% protein or more and seeds with over 45% protein on a dry weight basis (Benson Hill, Inc 2021).

Depending on how soybeans are grown, for instance, in wild or cultivated conditions, their protein composition changes. Moreover, genetic modifications can also change the way soy proteins express themselves. Albumins are dissolvable and globular proteins, which are alkali, and are two of the four polypeptide groups found in soy protein. Soybean is primarily composed of globulin proteins. It is possible to separate two important storage proteins, glycinin (11S), which is 30% soy protein, and conglycinin (CG, 7S), which is 40% soy protein, by ultracentrifugation (Natarajan et al. 2013) (Sui et al. 2021).

In seeds developed and matured from five special soybean genotypes, including two commercial cultivars Jack and Ozark, they were probed to determine the compositional change during seed development and maturation with regard to oligosaccharides, oil, protein, and seed size. Oil, protein, soluble saccharides, and starch were analyzed in seed samples taken every 7 days from initial seed formation until full maturity after approximately 8 weeks. Some compositional changes among the seven soybean genotypes followed similar trends, despite significant differences in their chemical compositions. During the first 3 to 5 weeks following flowering, protein content decreased and then gradually increased. As the process progressed, oil accumulated rapidly. Starch levels ranged from 6% to 15% in developing seeds but fell rapidly to 0.2%–1% once seeds reached maturity. A decrease in sucrose occurs during seed development and maturation, whereas nondigestible oligosaccharides (raffinose, stachyose, and verbascose) increase toward maturity. Specialty soybean varieties can be developed and selected based on these findings (Saldivar et al. 2011).

### 5.3 Carbohydrates

One significant class of storage chemicals found in soybean seeds is carbohydrates. There are two types of carbohydrates: soluble and insoluble. Because of their usefulness in food and feed applications, soluble carbohydrates have attracted greater attention than insoluble ones. The three primary components of the 15% soluble carbohydrates found in typical soybean seeds are sucrose (about 5%), raffinose (approximately 1.5%), and stachyose (approximately 5%) (Wang et al. 2014).

The primary source of metabolizable energy (ME) for animal feeds among these three major soluble carbohydrates is sucrose, which is also the most readily digested. A higher concentration of sucrose (3900 kcal/kg) is of importance to animal producers since it has a substantially higher ME than starch (2918–3396 kcal/kg) (Ostezan et al. 2023). Additionally, the sweetness of soy‐based goods, such as natto, tofu, edamame, and soymilk, is positively connected with increased sucrose concentration (Wang et al. 2023). Because of its better ME effectiveness for animal feed and the inherent sweetness of soymeal for human consumption, increasing the sucrose content has therefore become more common in soybean breeding projects (Ficht et al. 2022).

Raffinose and stachyose, the other two soluble carbohydrates, are referred to as raffinose family oligosaccharides (RFOs), which are antinutritional factors (ANFs). Raffinose synthase (RS) converts sucrose to raffinose by introducing galactose, which starts the chain elongation process. The second chain elongation is then carried out by stachyose synthase (SS), which creates stachyose by adding additional galactose to raffinose. The inability of the α‐galactosidase enzyme to break down the glycosidic bond between the elongated chains renders RFOs indigestible in animals with monogastric digestion (Chaudhary et al. 2015).

The lower stomach uses undigested raffinose and stachyose as a substrate for microbial fermentation, which releases carbon dioxide, methane, and hydrogen sulfide. This can lead to pain and diarrhea and ultimately reduced feed energy efficiency (Jo et al. 2018). Therefore, removing these ANFs along with improving sucrose in soybean seed is critical to improve ME efficiency for animal feeds as well as for market preference.

### 5.4 Fats

Global food security, agricultural output, and the economy as a whole are all impacted by soybeans (Liu, Li, et al. 2020; Liu, Zhang, et al. 2020). Global soybean oil production has increased significantly over the past 10 years, by about 44% (42.8–61.6 million Mt), as a result of growing public awareness of the environmentally friendly and healthful qualities of vegetable oils. Soybean oil has also been continuously highlighted in the biofuel industry and food applications. The fatty acid composition of soybean oil primarily determines its quality and functionality for use in industrial and food applications. The five main fatty acids found in soybean oil are palmitic (16:0, approximately 13%), stearic (18:0, approximately 4%), oleic (18:1, approximately 20%), linoleic (18:2, approximately 55%), and linolenic acids (18:3, approximately 8%) (Bilyeu et al. 2018).

For example, increased seed concentrations of polyunsaturated fatty acids (PUFAs) like linoleic and linolenic acid significantly decrease the oxidative stability and shelf life of soybean oil. A common method for lowering excessive PUFA content in soybeans is partial hydrogenation. However, this procedure produces trans‐fatty acids, which have been linked to adverse health outcomes, such as cardiovascular disease (CVD), when consumed (de Souza et al. 2015). High oleic acid (> 70%) soybean oil is also linked to improved nutritional value and oxidative stability without the harmful trans‐fatty acids produced by partial hydrogenation (Combs and Bilyeu 2019). Stearic acid content raises the melting point of fat, keeping it from melting at room temperature, which is crucial for the baking industry as well as for oxidative stability (Jeong et al. 2017). Consequently, using genetic methods to lower PUFAs and enhance the functionality of soybean oil without sacrificing any of its beneficial qualities has proven to be an effective strategy.

### 5.5 Other Constituents

Soy has a well‐established hypocholesterolemic impact; soy protein has been approved by regulators for lowering CVD risk. On the other hand, extra nutrients included in soybeans, like fiber, lecithins, saponins, and isoflavones, may enhance cardiovascular health via separate processes. Soy components that are not protein‐rich are shown to have cardiovascular benefits, which is compiled in this review with respect to established CVD risk factors, including inflammation, obesity, hypertension, and hyperglycemia, in addition to cholesterol lowering.

**FIGURE 4:**
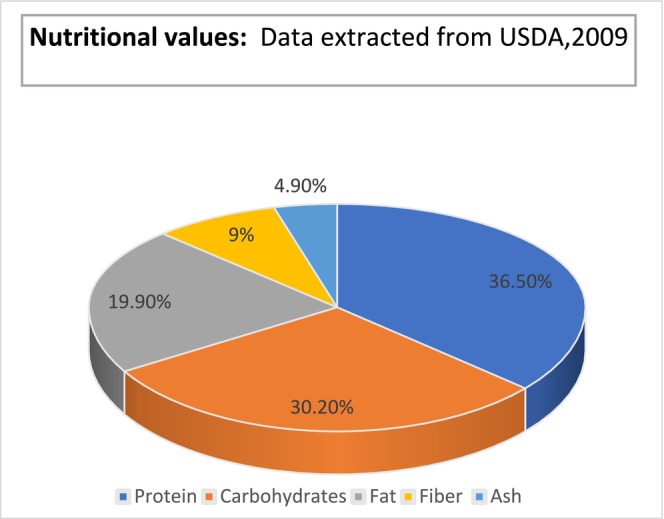
Chemical composition of soybean seed.

All things considered, the data to present indicates that non‐protein soy elements enhance cardiovascular health markers; yet, further meticulously planned research is needed to independently clarify these benefits. Additional research is required to elucidate the impact of the composition of the gut microbiota and the isoflavone‐metabolizing phenotype on the biological effect (Ramdath et al. 2017). Ninety‐two percent of the polysaccharides found in soybeans are found in cell wall material (Table 5, Figure 4).

**TABLE 5:** Nutritional values or chemical composition of soybean data extracted from USDA 2009.

Nutrients	Percentage
Protein	36.5 (but recent reports suggest it can vary)
Fiber	9
Fat	19.9
Ash	4.9
Carbohydrates	30.2

## 6 Production

Mississippi's economy relies heavily on soybeans, which are a major crop. Helms and Watt (1991) argue that soybean yields should be increased due to their nutritional and industrial properties.

### 6.1 Impact of Broiler Litter on Soil Nitrogen and Phosphorus Concentrations on Soybean Production

In addition to symbiotic N2 fixation, soybeans [ *Glycine max* (L.) Merr.] may benefit from supplemental nitrogen from broiler litter. A Leper salty clay loam (fine, semiotic, nonacid, thermic Vertis Epiaquerts) at the Mississippi Agriculture and Forestry Experiment Station in Starkville, MS, was used to evaluate the effects of broiler litter and commercial fertilizer applications on soybean yield, N and P uptake, and residual soil N and P. Plant‐available N (PAN) was applied to broiler litter at a rate of 0, 40, 80, 160 kg/ha alongside commercial fertilizer applied at PAN and P rates comparable to broiler litter. In relation to the amount of broiler litter and commercial fertilizer applied, soybean grain yield and nitrogen and phosphorus uptake quadrupled. In contrast to commercial fertilizers, broiler litter applications resulted in higher yields and nitrogen uptake in soybeans. A linear increase in soybean grain yield is associated with increasing nitrogen uptake, not soybean P uptake. There is an apparent recovery value of 80 kg PAN when broiler litter is applied. According to the declining data, soybeans were not able to effectively use increasing soil NO3‐N and P accumulations at the top 15 cm of the soil profile. Broiler litter treatment increased grain yield by 3.4% for every unit of nitrogen uptake. Broiler litter may benefit soybeans, according to this study (Adeli et al. 2005).

### 6.2 Impact of Soybean Harvest Time on Seed Quality, Yield, and Growth

It is less efficient to grow, develop, and yield soybeans [ *Glycine max* (L) Merr] in unfavorable environments. A delay in planting affects plant growth, leaf area index, normalized difference vegetation index, and grain yield as a result of changes in photoperiod, temperature, and precipitation. Delayed planting can also affect soybean seed quality. Extreme heat and drought can adversely impact plant growth and yield, causing a delay in planting. As a result of heat stress, a reduction in photosynthetic activity decreases the size and yield of seed sets. When plants are stressed during reproductive stages, they exchange carbon dioxide, photosynthesis, sugar, and metabolites. As a result, flower and pod abortion increases, and vegetative growth and seed number and size are reduced, as well as the duration of seed filling. With the combination of photoperiod, temperature, and precipitation, and delayed planting, soybean grain yields are likely to be reduced, as well as photosynthesis and plant growth (Hu and Wiatrak 2012).

### 6.3 Implications of Planting and Maturity Dates for Early Soybean Production System on Shattering Patterns

The Midsouth's early soybean production system (ESPS) uses mostly maturity group IV soybeans, which are prone to seed shattering. Genetic studies of soybean shattering resistance have been carried out for a number of varieties; however, there is little information about how soybeans shatter under specific environmental conditions, which is often critical to soybean growers. The shattering patterns of 80 to 132 MG IV soybean varieties were investigated at Stoneville, MS, on a yearly basis from 2007 to 2009. Historically, most soybean varieties did not shatter within the first 3 weeks after maturity (WAM) when April‐planted MG IV soybeans matured in mid‐to‐late August. Four WAMs revealed that pod shattering differed among varieties. Plants planted late in September matured in early September and showed low shattering rates, with seeds holding up to 6 WAM before shattering. Pod shattering was not significant in most soybean varieties planted in April 2009. Shaping began at 6–7 WAM. April‐planted MG IV soybeans are likely to suffer from delayed shattering due to relatively low temperatures and abundant rainfall in 2009. Soybeans planted in May 2007 and April 2009 showed fewer shattering problems when they matured after September. The seed shattering pattern could be affected by seasonal differences in weather patterns, especially temperature and rainfall (Zhang and Bellaloui 2012).

## 7 Genetics

### 7.1 Genetic Modification

Genetically modified crops first appeared in the market in 1994. Their acreage now makes up the majority of several key crops, such as maize, soybean, cotton, and canola, due to their steady and rapid acceptance, particularly in North and South America (Duke and Cerdeira 2010; Ronald 2011). With the advantages of increased yield, reduced input costs, and better environmental profiles, a growing number of transgenic traits are being used to increase herbicide resistance and insect resistance (Ronald 2011; Cerdeira et al. 2011).

The concern of whether genetically modified crops are as safe to eat for humans and animals as traditional crops has always been the most significant—and frequently the most contentious—aspect of the acceptance of genetically modified crops. This also pertains to the crops' effects on the environment. In order to obtain regulatory authorization for commercial release, broad composition as well as functional testing has always been necessary. Thus far, this concept has proven successful; after over 10 years of broad adoption, human and animal health do not appear to be affected by genetically modified crops and their deep incorporation into food and feed chains (Cerdeira et al. 2011).

Undoubtedly, sustained prosperity necessitates specific proof that every novel genetically modified product satisfies comparable or enhanced criteria. As a test strategy, the most popular is to compare the GM line with its closest isogenic version. Experience has demonstrated that, with the anomaly of the variable pertaining to the specified transgenic characteristic, transgene impacts on plants for traits that are widely used today are typically minimal. In fact, studies conducted over a number of years at various locations have demonstrated that environmental factors can have a far bigger impact on the overall metabolic variation within a given line than the variation resulting from the transgene itself (Baker et al. 2006; McCann et al. 2005).

Furthermore, there might be a wide range of genetic diversity found in commercial germplasm carrying modified traits. A single genetically modified crop line carrying a trait resistant to pests or commercially viable herbicides could not be produced outside of that genetic zone. There are individual, typical commercial crop lines that are designed for a wide range of regional conditions. Because of this, it is necessary to introgress the GM trait into a variety of formal cultures. Consequently, when comparing transgenic crops with germplasm expression, it is imperative to consider their variability.

Legislative bodies rightfully expect that the most sophisticated and precise technologies be employed in the evaluation of safety and equivalency for genetically modified agriculture (GM) despite the wide range of approaches utilized in previous studies. The prevalent methods during the initial 10 years of genetically modified agriculture (GM) regulation comprised comprehensive compositional and performance evaluations for attributes like specific analyses that were conducted for amino acids, fatty acids, secondary metabolites, and recognized toxins and antinutritive compounds; digestibility (for animal feed); and gross levels of total protein, starch, fiber, fat, and other compounds (Novak and Haslberger 2000; Aulrich et al. 2001).

Investigation was expanded to include non‐directional “profiling” techniques as analytical technologies developed (Kuiper et al. 2002). This was justified by the idea that such analysis could provide information about the potentially harmful effects of transgene expression as well as unpredictable pleiotropic effects (Cheng et al. 2008).

In one instance of the latter, unfavorable changes in expression may be connected to the gene insertion site, which is typically a random location in the genome. Proteomics (Corpillo et al. 2004), NMR‐based metabolite fingerprinting, HPLC or GC/MS metabolite analysis (Baker et al. 2006; McCann et al. 2005), and global gene expression profiling (Cheng et al. 2008) were among the profiling techniques that were quickly being used.

We have moved from unintended worldwide metabolomics analysis to more sophisticated and informative technologies that have been developed to study GM crops; you will need to evaluate them more carefully. The worldwide examination of small molecule metabolites, or “metabolomics,” has proven to be an effective and sensitive technique for identifying changes in the metabolic makeup of plants.

We identified 169 metabolites across a variety of metabolic pathways and classes after analyzing seeds from 49 standard soybean lines. The metabolome of natural soybean seeds was shown to have dynamic ranges based on parallel quantities of metabolites listed. With the exception of the designed pathway, the metabolome of a soybean plant's General Modified line, for instance, was found to be well within the natural variance when we examined it in more detail (Clarke et al. 2013).

## 8 Oil Extraction

Global demand for oils, fats, and lipids is rising, according to market analysis. In addition to being utilized in the food industry to produce edible goods, oils and lipids are also a key ingredient in a variety of non‐food products, including paints, waxes, varnishes, lubricants, soaps, synthetic resins, oils, and greases (Boulard et al. 2015). To extract the oil from seed or nut materials like rapeseed, sesame seeds, sunflower seeds, almond nuts, or hazelnuts, an extraction procedure is required. Cold pressing, hot pressing, and solvent extraction are the conventional ways for extracting oil; the former is a mechanical process, while the latter is a chemical one (Geow et al. 2018). Only around 80% of the oil in oleaginous material can be recovered using physical methods; therefore, alternative technologies must be used to recover the remaining 20% (Puértolas et al. 2016).

As technology has advanced over time, the conventional extraction process has been refined to increase oil yield or produce high‐quality end products. The process of removing oil from the sample, whether by mechanical or chemical means, remains the same; nevertheless, before being categorized as a marketable commodity, the oil that is extracted will go through a number of treatment procedures. To create edible oil of a suitable grade, those procedures include deodorizing, bleaching, neutralizing, and degumming. In those procedures, the contaminants will be eliminated chemically or physically to stop the oil from becoming unstable while being used by the user. The extraction process affects the oil's quality. A number of factors, including economic considerations, method efficacy, and environmental impact, influence the choice of extraction technique.

Balancing the economic and environmental aspects of edible oil production has recently been a top priority for the food business. Customers who are more aware of sustainable food production and its three pillars—people, planet, and profit—have been the primary force behind this change. More research has concentrated on enhancing extraction techniques to use less energy and produce fewer chemical pollutants as a result of the growing awareness of the environmental effects of unit operations employed in processing and the growth of green chemistry (de Oliveira et al. 2013).

### 8.1 Conventional Oil Extraction Processes

Simple mechanical devices that are controlled by hand or animal are used in the conventional way. These devices, which are frequently locally designed, mill oilseeds such as mustard, copra, soybean, peanut, and so on in rural areas without the need for fuel or power. These machines' expulsion capacity is significantly lower because they are manually operated (Sheikh and Kazi 2016). The traditional techniques consist of mechanical pressing and chemical or solvent extraction. These are the most extensively used and well‐liked techniques for extracting oil.

### 8.2 Mechanical Pressing

Seeds with more than 20% oil content are frequently pressed mechanically to remove the oil. Based on mechanically compressing seeds, it consists of two stages: preparation and extraction. In order to prepare seed material for oil extraction, it must be cleaned, broken, ground, and cooked (Sinha et al. 2015). After the seed material has been cleaned, the cell wall is broken down by rolling, flaking, and grinding. After being cooked at 90°C to 115°C, the mixture is further processed using a hydraulic press that is powered by fluid pressure. Compared to conventional oil processing techniques like rendering, the high pressure used in hydraulic presses yields a noticeably larger oil yield. When combined with the screw press method, which offers somewhat greater yields than the hydraulic press method, the hydraulic press method may be further enhanced. The horizontal and vertical screws are inserted into a perforated cage in a screw press. To increase the pressure on the material, the screw and cage are tapered toward the discharge. The pressure created determines how much oil is extracted by screw pressing. In contrast to the hydraulic press, where the applied pressure can be controlled or regulated, the screw press's pressure is harder to forecast and control (Bogaert et al. 2018). Several researchers have used screw presses to extract oil in order to achieve a high oil output (Chouaibi et al. 2019; Subroto et al. 2015). In rural locations, mechanical pressing is typically chosen because it requires less capital and is simple enough for semi‐skilled workers to use. The phosphorus, acid, iodine, and water contents of the oil that is mechanically extracted are typically used to gauge its quality (Subroto et al. 2015).

Its main drawback is that mechanical pressing is ineffective, leaving 8%–14% of the oil in the cake (Ali and Watson 2014). This method is typically used in conjunction with solvent extraction to extract the residual oil from the meal. Furthermore, it is labor‐intensive (Bhuiya et al. 2020) and time‐consuming, requires high pressure and temperature to function, reduces oxidative stability, breaks down valuable oil components, and diminishes oil quality because of the heat produced by expeller pressing (Yusoff et al. 2016).

Oil extraction can be carried out at low temperatures, known as cold pressing (below 50°C), to get around the issues caused by high heat. Due to low‐temperature pressing or extraction, cold‐pressed oils are of higher quality and retain their inherent qualities, distinctive flavor, nutritional profile, and purity (Kittiphoom and Sutasinee 2015). In order to keep the temperature below 40°C, dehulling the seeds is an essential step before cold pressing. In contrast to the hot‐pressing method, which extracts up to 80% of the oil present in seed, the cold‐press method delivers high‐quality oil but has a lower extraction yield because of the decreased oil viscosity at high temperatures (Patel et al. 2016).

### 8.3 Solvent Extraction

This technique is widely used to recover oil from medium‐ and high‐oil‐containing seeds (soybean and cottonseed) as well as high‐oil‐containing seeds (rapeseed, sunflower, and peanut seeds). This method is used in the large‐scale food sector to produce vegetable oil (Matthäus 2012). Numerous organic solvents, including ethanol, acetone, pentane, n‐hexane, chloroform, and ethyl acetate, are used in solvent extraction. Two criteria—very low viscosity and selective solvent—are used to determine which solvent is best for this process of oil extraction.

#### 8.3.1 Solvent Extraction Using Hexane

Hexane's superior qualities, including its easy recovery from the extract, low latent heat of vaporization (330 kJ/kg), narrow boiling point range (63°C–69°C), excellent solubilizing ability, high extraction efficiency, and nonpolar nature, make it the most widely used solvent for oil extraction worldwide (Kumar et al. 2017). The most widely consumed vegetable oil in the United States, soybean oil, is specifically made via hexane extraction (American Soybean Association 2019). Low production costs and great oil recovery are offered by this technique. In addition to being a hazardous air pollutant, hexane is extremely poisonous and combustible, despite the fact that its use in the industry offers benefits such as producing a defatted meal with a tolerable odor and little residual oil content (Toda et al. 2016).

The type of pretreatment used for the oilseed, including reaction temperature, the type and nature of the solvent (polar and nonpolar), the solid/solvent ratio, the reaction between the kernel and the solvent, and reaction time, all affect the quality of the crude oil and meal produced by solvent extraction (Mas'ud et al. 2017). According to one study, n‐hexane (37.03%) yielded greater extraction yields from sesame seeds than acetone (4.37%) and chloroform (6.73%) (Osman et al. 2016). One study, based on historical scenarios from 1980 to 2014, provided a techno‐economic evaluation of soybean oil extraction using hexane. According to their findings, the process becomes economically viable when soybean oil production exceeds 34.64 × 103 tonnes annually, and the production unit's economic viability makes more sense when considering capital costs and soybean prices (Cheng and Rosentrater 2017).

Free fatty acid values were comparable in both solvent extracts of soybean oil but marginally higher in ethanol extracts of palm and coconut kernel oils, according to a different study that performed phytochemical analysis on oils extracted from soybean seed, palm kernel seed, and coconut using ethanol and hexane as solvents (Chioma et al. 2020). The ethanol‐extracted oils were found to have greater acid and saponification values. According to the studies, hexane yielded a higher extraction rate than ethanol. The authors proposed ethanol as a preferable solvent for oil extraction since it is safer, more effective, and environmentally friendly, even if n‐hexane produced a higher extraction yield.

#### 8.3.2 Solvent Extraction Using Ethanol

Hexane substitutes have been studied, including ethanol, a natural, non‐toxic solvent that is permitted in the manufacturing of organic food (Baümler et al. 2016). Ethanol can extract more polar substances, including polyphenols, pigments, and soluble sugars, since it is more polar than hexane. When sunflower collets (ground oilcake or expanded material) were extracted, the advantages of utilizing ethanol over n‐hexane were shown, with a 32% vs. 23% yield of extracted material (oil and other chemicals), respectively (Sbihi et al. 2018). Recent studies have focused on ethanol as a solvent for soybean oil extraction. One study examined the effects of water levels (0%, 6%, and 12% weight percent of water) and temperature (varying from 40°C to 90°C) in this system. Their findings demonstrated that while an increase in water content had a detrimental effect on extraction, an increase in temperature improved oil removal (Sawada et al. 2014).

The most effective method for recovering oil is solvent extraction. Some organic solvents, however, are unfit for human consumption and have a negative impact on human health. Since medical and toxicological investigations have shown no negative effects on human health over an extended length of time, the United States Food and Drug Administration (USDA) has listed solvents (such as ethanol and acetone) as generally recognized as safe (GRAS) (Food and Drug Administration (FDA) 2016; Molino et al. 2018).

**FIGURE 5:**
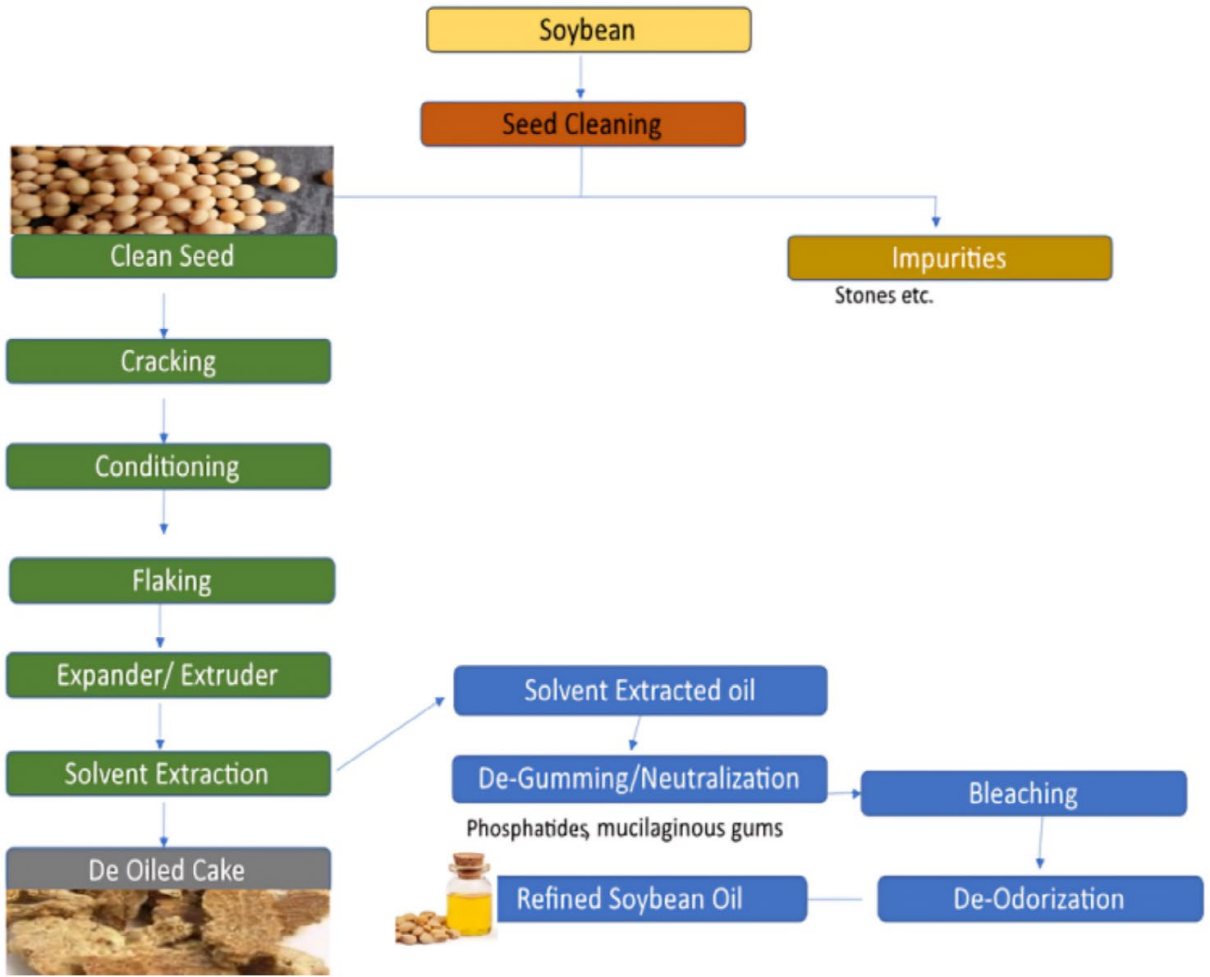
Process flow chart of soybean oil extraction through solvent extraction method.

**FIGURE 6:**
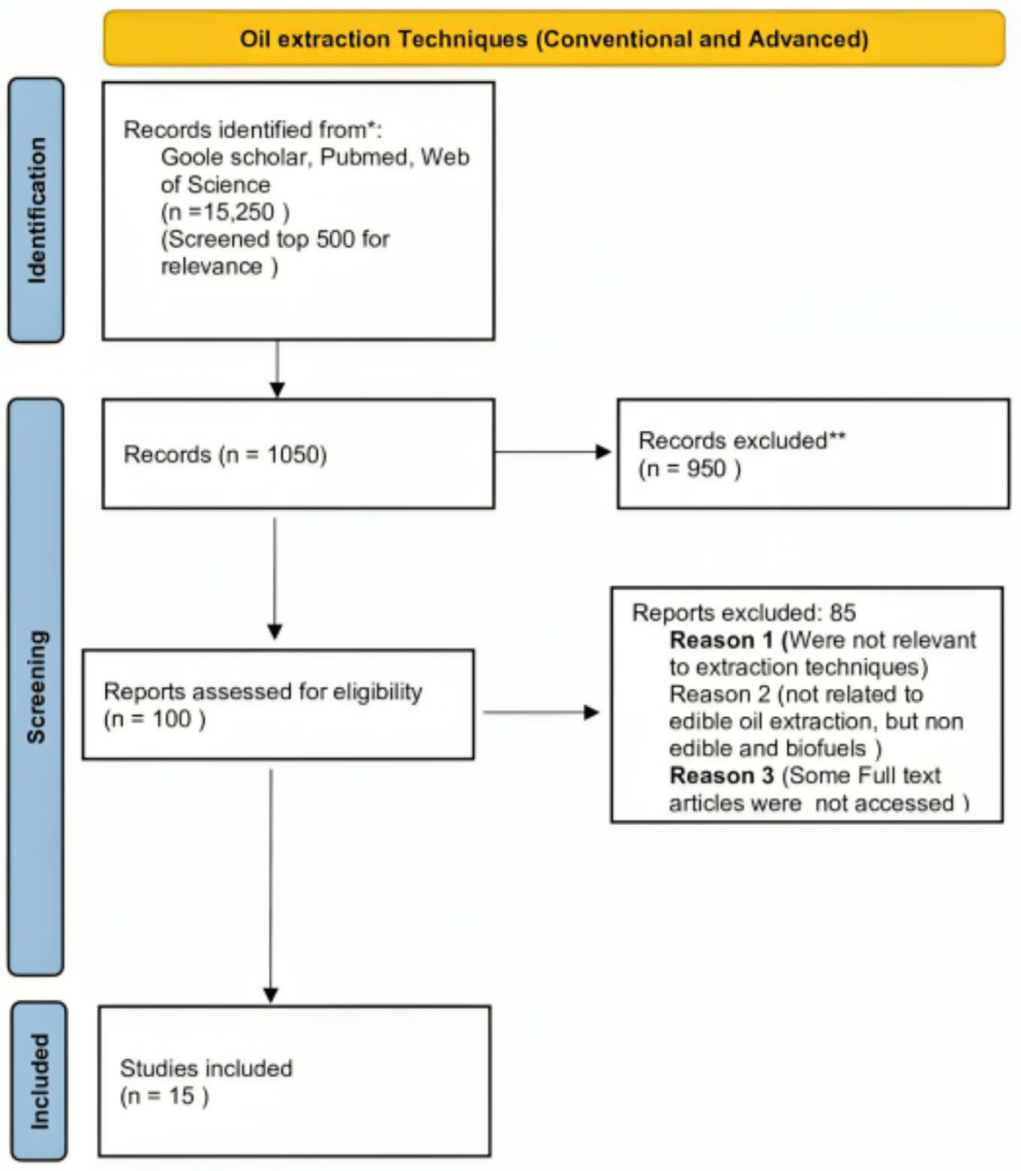
PRISMA flow diagram of oil extraction techniques.

As shown in Figure 5, a basic flowchart of a process that combines pressing and solvent extraction to extract oil from oilseeds with high oil content (Figure 6).

However, solvent extraction costs a lot of money, energy, solvent consumption, and operating expenses. It also takes a long time to complete, involves many processing processes, and degrades the quality of the oil because of the heat produced at high temperatures (Takadas and Doker 2017). Advanced oil extraction technologies have had to replace solvent/chemical‐based technologies due to the harmful effects of chemicals on human health and the environment, as well as other difficulties presented by traditional techniques of obtaining high‐quality oil from plants.

## 9 Advanced Oil Extraction Techniques

Enzymatic‐assisted extraction (EAE) and ultrasound‐assisted extraction (UAE) are two of the more sophisticated solvent‐coupled methods for oil extraction. These methods are thought to be the way of the future for the oil extraction sector since they are environmentally friendly, produce a large amount of oil, use less energy, and command a premium price on the market.

### 9.1 Enzyme‐Assisted Extraction

An EAE technique for oil extraction has been developed, which is easy to use and uses little energy to combat the low yield issues with mechanical pressing and the harmful environmental effects of solvent extraction (Yusoff et al. 2016). There are several steps involved. In order to facilitate the simple diffusion of the oil into the extraction medium, the oilseeds are first roasted and then transferred to a container of water to which enzymes are introduced to aid in the digestion of the solid cell material. The remaining enzymes are then extracted from the oil using a liquid–liquid centrifuge. High‐value goods are recovered by EAE. Oil companies use this technique because it works at low temperatures and is matrix (tissue) specific. According to a study, a high oil recovery of 87.58% and a very fragrant oil were obtained when sesame oil was extracted using enzyme‐assisted aqueous extraction (trypsin, papain, and cellulase) (Hou et al. 2013).

A crucial first step is choosing the right enzyme or combination of enzymes. Cellulolytic, hemicellulolytic, proteolytic, phospholipase, and pectolytic enzymes are the most commonly used enzymes for cell wall digestion. The target oilseed's tissue structure and the operational environment are taken into consideration while choosing the enzyme combination (Nde and Foncha 2020). According to one study, there are a few benefits to using enzyme‐assisted aqueous extraction for soybean oil extraction, including low acid and peroxide values (de Moura Bell et al. 2013).

### 9.2 Ultrasound Assisted Extraction

In order to help extract components of interest from plant sources, ultrasound has been extensively researched in the food business (Chandrapala et al. 2013). Ultrasonication's mechanical action uses ultrasonic waves (20–100 kHz), which cause the cell wall to collapse and release the soluble chemicals. For a number of reasons, including shorter extraction times, the use of solvents, and more efficient energy use, as well as better product quality, ultrasound has been used and shown promise as a green technology in the extraction industry (Chemat et al. 2017).

Cavitation is the process by which the liquid's existing pressure drops below its vapor pressure during extraction using ultrasonic sound waves. This results in the formation of a bubble, which expands and contracts in response to changes in the applied pressure and frequently collapses, damaging the cell structure and releasing its contents. Additionally, acoustic cavitation stirs the solvent, allowing for increased penetration into the cell matrix (Danlami et al. 2014).

According to one study, oil yield rises with ultrasonic power; however, if the power surpasses 90 W, oil yield sharply declines because cavitation disrupts the oilseed's molecular structure, lowering the extraction rate. The effectiveness of UAE depends on temperature, time, ultrasonic frequency, and solvent type and concentration (Wang and Wei 2015). Water is used as the most common solvent in the UAE because a rise in vapor pressure and surface tension of the solvent decreases the intensity of cavitation. The oil output can be increased by combining conventional techniques with ultrasound pretreatment. The UAE's main benefits are its ease of use, affordability, environmental friendliness (Hashemi et al. 2015), low energy consumption, quick extraction time, increased solvent penetration, low solvent requirements, and high oil yield (Ayim et al. 2018).

One of the main problems with UAE technologies is attenuation, which is the process wherein the amplitude and intensity of ultrasonic waves diminish with distance. Actually, only a small area close to the ultrasonic emitter is the activated ultrasound zone (Danlami et al. 2014).

## 10 Conventional vs. Advanced Oil Extraction Techniques

These oils and fats are currently being extracted using both traditional and innovative techniques, either on a large scale or as pilot projects (Tiwari 2015). Nonetheless, the oil extraction industry is dominated by traditional methods, including solvent extraction and mechanical expression. Because existing technologies have several drawbacks, such as higher energy costs, longer turnaround times, lower yields, and less environmental friendliness, new procedures are required (Sharma et al. 2019).

Without sacrificing the quality of the oil or the oil recovery process, green solvents have a great deal of promise to replace the widely used n‐hexane. According to research, the drawbacks of standard methods for removing important components from plants and seed materials have been successfully and efficiently overcome by unconventional extraction approaches. The higher quality of the extracted goods is what makes these methods better than traditional ones. Their time efficiency and solvent use are both reduced. Additionally, they produce a high yield, are economical, are environmentally benign, and allow for the production of co‐products without sacrificing quality (Chemat et al. 2017).

More research has concentrated on enhancing extraction techniques to use less energy and produce fewer chemical pollutants as a result of the growing awareness of the environmental effects of unit operations employed in processing and the growth of green chemistry (de Oliveira et al. 2013).

When choosing an extraction technique to use on an industrial scale, economic viability is the primary determinant, but environmental effects are also becoming more and more important. Oil extraction has been the subject of techno‐economic analysis (TEA), which breaks down costs and profits for any kind of industrial activity. When executing TEA, a wide range of factors must be taken into account, such as the location of the extraction plant, the type of substrate, and the scale of the extraction processes (Cheng and Rosentrater 2019). For assessing facility scale‐up and the ensuing economics of oil extraction operations, TEA is an effective technique. For instance, a TEA model on the extraction of hexane from soybean oil revealed that in order for the process to be financially viable, a plant capacity of 34.6 million kg of soybean oil output per year was required (Cheng and Rosentrater 2017).

When choosing which of the several extraction techniques now in use to employ, a number of considerations should be made, including profitability and environmental sustainability. Better extraction techniques backed by environmental effect and techno‐economic research would be advantageous for the specialty oil business as consumer interest in sustainable food items grows.

## 11 Sustainable Practices in Soybean Production and Processing

**FIGURE 7:**
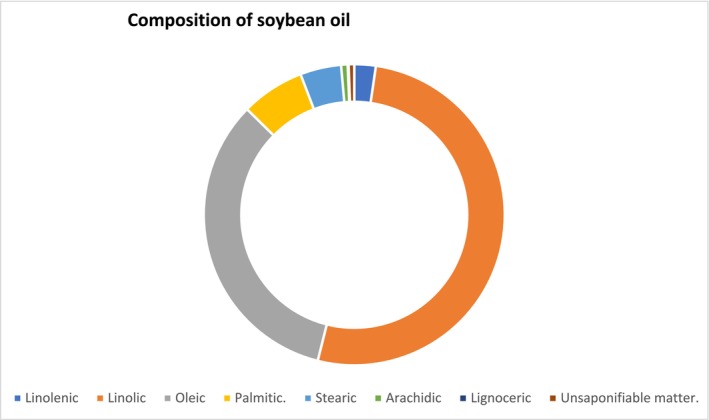
**C**omposition of soybean oil.

For soybean growers in the United States, sustainability is a top concern. Farmers use sustainable soy farming methods such as crop rotation, reduced‐till or no‐till, water and nutrient management, precision farming technologies, and cover crops as stewards of the land. These methods assist farmers in increasing crop yields, increasing efficiency, and producing sustainable soy (SoyConnection 2023). One of the biggest sustainability verification programs in the agriculture sector is the Soy Sustainability Assurance Protocol (SSAP), which has shown itself to be a very accurate indicator of U.S. soy farmers' dedication to topics like biodiversity, sustainable production methods, worker and public health, and ongoing farming practice improvement (USSEC 2023). Field to Market measures the sustainability of U.S. soybean production by looking at greenhouse gas emissions, soil erosion, energy, water, and land use. In the United States, soybeans have used 48% fewer acres per bushel, 60% less irrigation per bushel, and 46% fewer British Thermal Units (BTU) per bushel of energy on average since 1980. In terms of pounds of CO_2_ and tonnes of soil erosion per bushel, greenhouse gas emissions and soil erosion have decreased by 43% and 34%, respectively. Crop yields in tonnes per acre, meanwhile, have risen by 130% (CME Group 2025) (Figures 6 and 7, Table 6).

**TABLE 6:** A systematic analysis of soybean oil composition (Baughman and Jamieson 1922).

Acid	%
Linolenic	2.3
Linolic	51.5
Oleic	33.4
Palmitic	6.8
Stearic	4.4
Arachidic	0.7
Lignoceric	0.1
Unsaponifiable matter	0.6

## 12 Health Effects

### 12.1 Soybean Intake, Cholesterol, and Heart Disease

In both industrialized and developing nations, CVD is a major cause of illness and mortality (Saeed et al. 2017). Epidemiological studies show that CVD causes 597,689 deaths in the US each year and 13.2 million deaths worldwide. The health care system bears a heavy load and expense as a result of CVD. As a result, finding effective CVD prevention measures is essential (McAloon et al. 2016). Prior research on the association between diet and CVD has mostly examined food group intake, with little focus on nutrients, especially phytochemicals (Blekkenhorst et al. 2018). One kind of phytoestrogen present in soybeans and soy products is called soy isoflavones. Researchers studying CVD have long been interested in a class of phytochemicals called isoflavones. The majority of these polyphenolic chemicals are present in soy and its derivatives (Nachvak et al. 2019). Soy isoflavones have been shown to provide a number of cardioprotective advantages, including lowering low‐density lipoprotein cholesterol (LDL‐C), preventing platelet aggregation, cell adhesion proteins, and pro‐inflammatory cytokines, increasing nitric oxide generation, and enhancing vascular responsiveness (Zahradka et al. 2013). There is conflicting epidemiological evidence about the relationship between soy isoflavone intake and the risk of CVD, despite the fact that a number of mechanisms have been put forth (Im and Park 2021; Nozue et al. 2020). Higher consumption of soy isoflavones may significantly lower the risk of CVD or coronary heart disease (CHD), according to certain observational studies (Ma et al. 2020); however, other research has not identified any such correlations.

Two meta‐analyses found no evidence of a significant correlation between the risk of CVD and soy isoflavone intake (Yan et al. 2017; Lou et al. 2016). It should be mentioned that these meta‐analyses merged data from case–control and cohort studies, which led to inaccurate conclusions. Furthermore, those meta‐analyses provided little detail regarding the magnitude and nature of the dose–response relationship between soy isoflavone intake and CVD risk.

Protein isoflavones found in soy have improved cardiovascular indicators and reduced the risk of coronary heart disease by 27% during a 10‐year period, the myocardial infarction rate by 37%, the CVD by 24%, and the heart disease mortality rate by 42%. Additionally, soybean protein has anti‐atherosclerotic and anti‐obesity qualities by positively influencing cholesterol levels (Sathyapalan et al. 2018). A study looked into how the incidence of CVD was affected by fermented soybean products supplemented with okara. The trial was randomized and placebo‐controlled. Two groups of males with normal cholesterol levels were created. There were eighteen males (*n* = 18) in one group. Lactis Bb‐12, La‐5 ( *Lactobacillus acidophilus* ), and subsp.: Bifidobacterium were administered daily together with 100 g of fermented soy product. A dose of 100 g of unfermented soy product was administered to the second group. The trial had an 8‐week duration and included a placebo group. According to the findings, men with normal cholesterol had minimal CVD risk factors and a limited lipid reduction effect from soy products containing okara (Bedani et al. 2015). After reviewing the data on soy protein and cholesterol reduction, the FDA determined that 46 trials no longer support an unqualified (i.e., significant scientific agreement) heart health claim (Food and Drug Administration 2017). Although soy meals were known to be low in cholesterol, saturated fat, and omega‐3 fatty acids, the 3% decrease in LDL cholesterol that may not have been statistically significant was not regarded as clinically important. This decrease happens when soy products replace popular protein sources in the diet, like dairy and meat that contain saturated fat. According to estimates, displacement will further lower LDL cholesterol by about 4%. The relatively insignificant 3% decrease in LDL cholesterol was not regarded as clinically important, despite the fact that soy meals were known to be low in saturated fat, cholesterol, and omega‐3 (*n*–3) fatty acids (Blanco Mejia et al. 2019). The slight decrease in LDL cholesterol that soy offers, however, is comparable to the allowed claims for other plant foods and food ingredients (such as nuts, viscous fibers, plant sterols, etc.). Another benefit of soy protein and meals is that they can lower LDL cholesterol by up to 29% when combined with nuts, viscous fiber, and plant sterols/stanols (Tree Nut Nutrition Research and Education Foundation 2018).

Rats in a different recent study were fed a diet high in sucrose (62.5%) for 4 months. For 4 months, half of them ate the same thing, and half were fed SRD (soya was used in place of casein). The trial's findings demonstrated that when soy was substituted for casein, dyslipidemia, glucose homeostasis, and insulin resistance improved (Oliva et al. 2017).

According to a study, soy protein and isoflavones were effective in lowering inflammatory markers (IL‐6), but they had no effect on blood lipid levels, which are thought to be a significant risk factor for coronary heart disease (CHD) (Mangano et al. 2013).

It is possible that products containing soy protein lower the ischemic heart disease risk and other conditions, moreover, by reducing cholesterol levels and health risks. It reduces CVD risk since vegan protein diets are low in fat and triglycerides (Nowbar et al. 2019; Welty 2020).

Although soy protein is associated with enhanced vascular endothelial function, recent clinical trials do not support the theoretical arguments for soy protein's potential to reduce CVD risk factors. Soy protein as a CVD risk factor preventer needs to be proven in further epidemiological studies. As a result, fermented soybean protein products are still recommended to reduce CVD mortality risk as part of a balanced diet. Additionally, exercise is essential for maximizing the benefits of these products, as well as a healthy lifestyle (Katagiri et al. 2020).

### 12.2 Soybean Intake and Cancer

Numerous studies suggest that eating a balanced diet can help avoid cancer. The relationship between soy consumption and cancer risk has been the subject of numerous prospective observational studies. Despite the fact that prior meta‐analyses found that soy consumption was inversely linked to 15%–37% decreased risks of cancer in several sites, including the stomach, prostate, and lung (Lu et al. 2017), no meta‐analysis has compiled the relationship between soy intake and total cancer risk. It is crucial to consider cancer as a whole in order to evaluate the prevention of cancer‐related morbidity and death in the general community as well as cancer patients. A number of biological processes, including a stronger antioxidant effect and decreased inflammation, have been suggested as explanations for the possible health advantages of soy, particularly isoflavones' ability to prevent cancer. It is commonly known that high quantities of reactive oxygen species can cause DNA damage, induce gene mutations, and either accelerate or decrease cell death, which can lead to mutagenesis and carcinogenesis. It has been proposed that soy and soy isoflavones can combat oxidative stress by modifying the expression of genes associated with cell proliferation and death and activating nuclear factor erythroid 2‐related factor 2 (Nrf2) (Zhai et al. 2012).

Increased dietary intake of isoflavone was consistently linked to a higher risk of overall mortality rather than cancer‐specific mortality in patients with breast cancer, according to a recent meta‐analysis of 28 prospective and retrospective cohort studies on breast, lung, prostate, colorectal, and glioma. This finding further reflected the possible prevalence of coexisting chronic diseases in cancer patients. Additionally, it should be mentioned that the proportionate benefit of dietary soy intake may be diminished if there is a higher prevalence of competing risk factors, such as the negative effects of obesity, physical inactivity, smoking, and excessive alcohol consumption (Micek et al. 2020). Despite soy's beneficial effects on cancer incidence, another study found no correlation between soy consumption and cancer mortality risk in either the general population or cancer patients. The disparities seem to be explained by the fact that cancer mortality and incidence are two rather different outcomes, with cancer mortality being significantly influenced by the treatment methods a person receives. It is generally known that cancer therapies can cause a variety of negative side effects, such as nausea, vomiting, altered bowel movements, and loss of taste and smell, all of which can make it difficult to consume, digest, or absorb soy (Hoq et al. 2019).

The native East Asian legume known as soy, soybean, or soya bean is widely cultivated for its edible beans. Largely because of the presence of isoflavones, the beans and their products have been shown to have positive benefits on cancer. A recent meta‐analysis of 30 papers found a strong statistically significant link between eating soy and a lower risk of prostate cancer (PCa). The incidence of PCa was low in populations that regularly eat soy products, such as those in China and Japan (Applegate et al. 2018).

**FIGURE 8:**
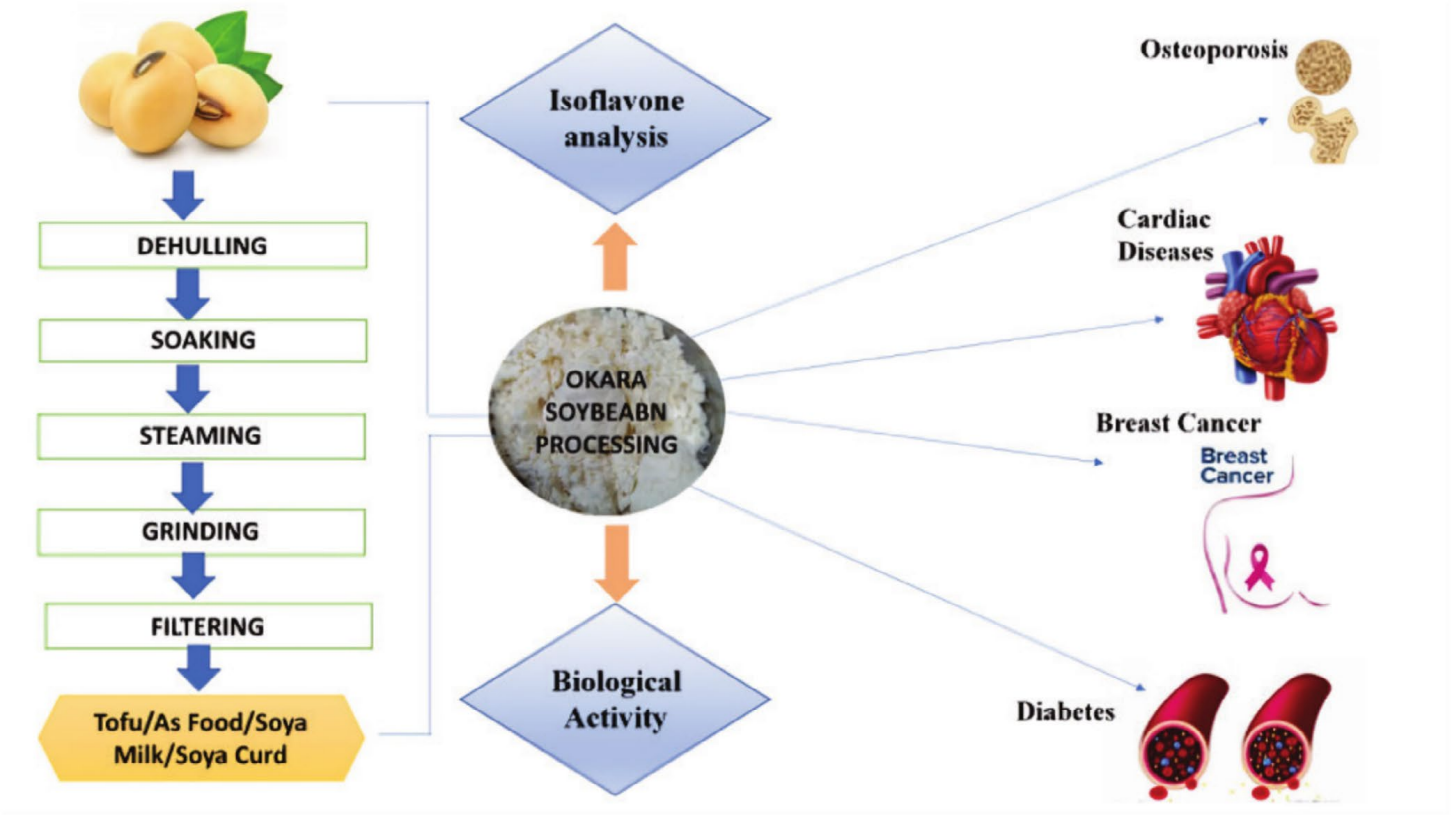
Therapeutic potential of soybean.

It's interesting to note that genistein has also been shown to be a strong inhibitor of angiogenesis and metastasis, which has promise for the prevention, management, and treatment of cancer. A possible function in combination therapy is suggested by the synergistic behavior of genistein with anticancer medications like doxorubicin, docetaxel, and tamoxifen (Spagnuolo et al. 2015). In animal models, genistein, a common isoflavone in soy, has been demonstrated to prevent the initiation, spread, and metastasis of cancer. It might work by altering the genes involved in apoptosis and cell cycle regulation (Zhang et al. 2013) (Figure 8).

### 12.3 Colon Cancer

One of the most prevalent cancers in the world, colorectal cancer (CRC) is the third leading cause of cancer‐related deaths (Inamura 2018). Soy isoflavones, particularly genistein, suppress cell growth and promote apoptosis and cell cycle arrest in the G2/M phase of human colon cancer. Quantitative polymerase chain reaction and immunoblotting show that ATM/p53, p21waf1/cip1, and GADD45α are activated along with cell cycle arrest in the G2/M phase, and that cdc2 and cdc25A are downregulated. Interestingly, genistein caused G2/M cell cycle arrest in a p53‐dependent way, indicating that the ATM/p53‐p21 cross‐regulatory network plays a critical role in mediating genistein's anticarcinogenic action (Zhang et al. 2013).

In a case‐control research, 925 CRC patients' dietary consumption data was collected using a semiquantitative food‐frequency questionnaire (SQFFQ). This survey contained five foods made from soy, a significant source of isoflavones, primarily genistein. The findings showed that consuming large amounts of soy products, which include isoflavones, can lower the risk of colorectal cancer (Shin et al. 2015). The relationship between the risk of colorectal cancer and important soy nutrients, such as phytoestrogens or isoflavones, was investigated in three meta‐analyses (Tse and Eslick 2014; Yu et al. 2016; Jiang et al. 2016). In one study, a meta‐analysis of 18 case–control and four prospective cohort studies evaluated the relationship between soy isoflavone consumption and the risk of colorectal cancer. According to their findings, consumption of soy isoflavone was linked to a lower incidence of colorectal cancer (CRC) in all groups. A subgroup analysis showed that these associations were strongest for soy meals and products in Asian populations. According to the investigators, eating soy isoflavones was most closely linked to a lower risk of colorectal cancer in Asian people (Yu et al. 2016).

### 12.4 Breast Cancer

Among women worldwide, breast cancer is the most prevalent type of cancer and one of the main causes of cancer‐related deaths (Bray et al. 2018). There is regional variation in its incidence rate, with Western nations having a far greater rate than Asian nations (DeSantis et al. 2015).

Dietary isoflavones, which are abundant in soy products, have structural resemblances to 17‐β‐estradiol and may act as effective estrogen antagonists to prevent breast cancer. Additionally, soy isoflavones may work through mechanisms unrelated to estrogen (Hüser et al. 2018). Several meta‐analyses found that Asian women, but not their Western counterparts, had a decreased incidence of breast cancer risk when they consumed more soy (Bahrom and Idris 2016).

There was no correlation between isoflavone intake and breast cancer, according to a recent meta‐analysis that only included cohort studies, which are less prone to selection and recall bias. However, it did find that consuming more soy‐based foods was marginally associated with a lower risk of breast cancer than consuming fewer soy‐based foods (Zhao et al. 2019). There was also conflicting evidence from research conducted in Asia, where dietary soy intake levels ranged from modest to high. While some studies found no statistically significant link, others found that women in the groups with the highest soy intake had a lower risk of breast cancer (Baglia et al. 2016).

The China Kadoorie Biobank (CKB) study found no correlation between soy intake and the incidence of breast cancer, with the highest consumption group's median daily intake of soy isoflavones being around 20 mg. This outcome was in line with the four cohorts that consumed a modest amount of soy (Morimoto et al. 2014). Women in the highest soy consumption group had a lower risk of breast cancer than those in the lowest consumption group in four studies where the highest quartile or quintile intake groups had soy isoflavone > 40 mg/day or the upper half intake group had a median intake of 23.5 mg/day (so the upper quartile group is likely to have < 40 mg/day) (Wada et al. 2013).

Although one study found that current soy isoflavone supplements were associated with an increased risk of ER‐breast cancer and a decreased risk of estrogen receptor positive (ER+) breast cancer, two Western studies that evaluated soy or soy isoflavone supplements did not find any association between soy or soy isoflavone supplements and overall breast cancer risk. Nevertheless, the investigation may find that the isoflavone dosage in the supplements varies (Touillaud et al. 2019). Researchers determined that a diet containing soy isoflavones would be safe for breast cancer survivors after discovering that they did not have estrogenic effects in people. These results imply that soy consumption may be chemoprotective and help patients with breast cancer avoid recurrence (Fritz et al. 2013).

A higher consumption of soy products is linked to a decreased chance of developing breast cancer, according to another study. However, after doing a meta‐analysis of data gathered from multiple prospective cohort studies, no meaningful correlation between an isoflavone‐rich diet and the risk of breast cancer was found. Therefore, more research is needed to determine whether consuming soy products lowers the risk of developing breast cancer (Zhao et al. 2019).

### 12.5 Prostate Cancer

As of 2020, prostate cancer (PCA) ranks sixth in terms of cancer‐related mortality and is the second most common cancer among males worldwide (Sung et al. 2021). It is projected that by 2030, there will be 1.7 million additional cases and 499,000 deaths from PCA, increasing the burden of the disease (Culp et al. 2020). There are still conflicting results from the research that has been done on the impact of soy products on PCA risk. Interestingly, there isn't a consensus on whether soy products affect PCA incidence.

Due to the complex makeup of soy products, their influence on the incidence rate of PCA cannot be explained by a single mechanism. According to accepted hypotheses, however, the prostate is protected by the high concentration of isoflavones (genistein, daidzein, etc.) found in soy products, which prevent the proliferation of PCA epithelial cells. By blocking angiogenesis during PCA growth and preventing cancer cells from adhering to blood vessel surfaces, studies have shown that isoflavones can efficiently and dose‐dependently suppress the growth of LAPC‐4 and PC‐3 PCA cells (Křížová et al. 2019). According to a recently developed viewpoint, soy products' ability to protect PCA is dependent on both their androgen‐like and anti‐androgenic properties. Prior studies emphasize the critical role that androgen levels play in the health of the male prostate, pointing to increased cellular androgen responsiveness or prolonged androgen exposure as important risk factors for PCA (Mahmoud et al. 2014).

At the moment, researchers are more inclined to focus on the anti‐androgenic qualities of soy products and the natural anticancer benefits of isoflavones than on the harmful effects of androgens on the prostate (Nakai et al. 2020). Natural androgens are found in soy products, and exogenous phytoestrogen supplements can cause hormone metabolism to change into androgens, increasing the amount of androgens in the blood and raising the risk of PCA (Ramírez‐de‐Arellano et al. 2022). Research has indicated that as PCA advances, cancer cells' sensitivity to hormone therapy may be weakened by mutations or epigenetic silencing of DNA repair genes, which would enable the shift from hormone‐dependent to hormone‐independent tumor growth. This phenomenon is especially important when treating patients with advanced PCA. This could be the reason why eating soy products has a protective effect on low‐grade or localized PCA but has no discernible effect on high‐grade or non‐localized PCA (Mateo et al. 2015).

In soybeans, genistein is one of the most abundant and potent flavonoids. PCa is one of the many biological processes that can change in estrogen‐related cancers. It has been discovered that it mostly works by changing angiogenesis, the cell cycle, and apoptosis while also preventing metastasis. The molecular mechanisms underlying genistein's anticancer and therapeutic effects have been proposed to include kinesin‐like protein 20A, B cell lymphoma 2 (Bcl2)‐associated X protein, Bcl‐2, extracellular signal‐regulated kinase 1/2, nuclear transcription factor κB (NF‐κB), mitogen‐activated protein kinase, inhibitor of NF‐κB, Wingless and integration 1 β‐catenin, phosphoinositide 3 kinase/Akt signaling pathways, and caspases (Spagnuolo et al. 2015). Its ability to inhibit NF‐κB is especially significant since it reduces inflammation. Cell survival and apoptosis are kept in a homeostatic equilibrium by the serine/threonine‐specific protein kinase Akt and the NF‐κB.

### 12.6 Inflammatory Bowel Disease

One chronic recurrent inflammatory condition is inflammatory bowel disease (IBD). Crohn's disease (CD), indeterminate colitis, and ulcerative colitis (UC) are the three types of IBD.

Recently, researchers have reported the effect of isoflavones on IBD. Glucosides and aglycones are the two types of isoflavones that the human body absorbs in the small and large intestines (Křížová et al. 2019; Franke et al. 2014). Glucosides can be hydrolyzed into bioactive aglycones in the proximal intestine for improved absorption, although isoflavones in the form of aglycones are absorbed more quickly. The majority of isoflavones in human blood are found as glucuronide (75%), followed by sulfate (24%) and aglycone (≤ 1%). Because of their structural similarities to estrogen, isoflavones can attach to estrogen receptors.

Studies on the impact of isoflavones on IBD have been published recently. Daidzein and glyceolins (isopentehexene isoflavone, a daidzein derivative produced by stressed soybeans) alleviated dextran sulphate sodium (DSS)‐induced colitis in a mouse model; as a result, they could be utilized to treat ulcerative colitis (Seo et al. 2017). UC patients benefited from a moderate isoflavone consumption during the remission phase. IBD is also impacted by soy isoflavones' estrogen‐like properties. It was discovered that estrogen improved the barrier function of intestinal epithelial cells (by up‐regulating the tight junction proteins), decreased the production of pro‐inflammatory factors through epithelial cells, and relieved endoplasmic reticulum stress (van der Giessen et al. 2019).

### 12.7 Osteoporosis

The most common long‐term metabolic bone disease is osteoporosis, which is caused by the intricate interplay between bone growth and resorption that is impacted by a number of variables. The World Health Organization classified osteoporosis in 1993 as a progressive systemic skeletal condition that increases bone fragility and fracture susceptibility due to decreased bone mass and microarchitectural degradation (Noh et al. 2020). There are two types of this condition: primary and secondary. Adolescent bone development abnormalities, disturbed bone remodeling in aging populations, and estrogen shortage in postmenopausal women can all contribute to primary osteoporosis (Amarnath et al. 2023). A number of conditions, including oxidative stress, metabolic abnormalities, chronic renal disease, and estrogen shortage, can cause secondary osteoporosis.

Classified as phytoestrogens, soy isoflavones share structural similarities with estrogen and may be a viable substitute. By acting as estrogen supplements, these isoflavones can lessen osteoporosis by easing menopausal and postmenopausal symptoms (Uehara 2013).

Research employing a mouse model to mimic weightlessness through hindlimb suspension revealed that soy isoflavones prevented bone degradation, inhibited the rise in the RANKL/OPG mRNA expression ratio, and stopped the unloading‐induced decrease in bone mineral density (BMD). Remarkably, this treatment greatly improved trabecular bone (Tousen et al. 2020).

## 13 Emerging Trends and Future Directions

### 13.1 Recent Developments in Genetic Modification Studies

Abiotic and biotic stressors are the two primary categories of stressors that impact soybean productivity. Viruses, insects, and nematodes are examples of biotic factors, whereas salt, drought, frost, heat, and flood are examples of abiotic forces. Transgenic techniques are employed to shield soybeans from these limitations.

The field of plant genetic modification has recently seen new potential due to the development of clustered regularly interspaced short palindromic repeats/CRISPR‐associated protein 9 (CRISPR/Cas9) technology (Shan et al. 2013). An effective method for developing crop cultivars with increased resistance to environmental stressors is CRISPR/Cas9 genome editing. A study showed that CRISPR/Cas9 genome editing enhanced soybeans' ability to withstand salinity by mutating the GmAITR genes. Gmaitr mutants showed improved salt tolerance throughout both the seed germination and seedling phases. Therefore, using CRISPR/Cas9 to delete the GmAITR genes was a successful method for making soybeans salt‐tolerant (Wang et al. 2021). One of the main obstacles to the development of soybeans is insect pests, such as leaf‐chewing insects. The creation of soybean cultivars that are more resilient to insects that consume leaves can significantly boost soybean yields in this situation.

Another biotic element that poses a significant risk to soybeans is nematodes. The crop is vulnerable to about 100 nematode species, which are a frightening menace and seriously harm soybean crops grown all over the world. In soybeans alone, soybean cyst nematodes (SCN) result in yield losses of over $1.5 billion annually. The primary means of management, crop rotation and the selection of resistant cultivars in the field, is insufficient to eradicate the nematode from soybeans entirely (Lund et al. 2018). To develop soybeans resistant to nematodes, transgenic techniques have been employed. Resistant alleles, including rhg‐1, were discovered that aid in resistance to SCN. Resistance to the soybean cyst nematode is provided by transgenic soybeans that overexpress the GmSAMT1 gene (Lin et al. 2016).

It has been observed that Agrobacterium genetic transformation of soybeans has a very poor efficiency. In such systems, promoters are helpful, but their efficiency and selectivity are quite poor. The CRISPR/Cas9 system benefits greatly from one such promoter system, germline‐based promoters. The quantity of chimaeras in converted soybeans is decreased by using germline‐based promoters. To increase the effectiveness of new plant breeding technologies (NPBT) in soybeans, the introduction of germline‐based promoters may be crucial (Mao et al. 2016).

### 13.2 Advances in Extraction Technology

In contrast to conventional solvent extraction techniques, green technologies are currently being used to create innovative extraction procedures that preserve safe and high‐quality extracts while utilizing nonhazardous solvents or renewable natural resources. It is more advantageous to use a novel extraction technique that uses less energy than the conventional approach. In order to increase throughput and decrease the use of organic solvents in the conventional solvent extraction process, the supercritical fluid extraction (SFE) method was introduced to the extraction industry (da Silva et al. 2016). In order to ensure safe and high‐quality extracts, green extraction has mostly concentrated on the development of innovative extraction techniques with lower energy requirements, using renewable natural resources and nonhazardous alternative solvents. Supercritical carbon dioxide, out of all the supercritical fluid options, is primarily utilized as an alternative solvent in food applications due to its affordability, cleanliness, and capacity to generate free‐solvent products (Akalın et al. 2016).

### 13.3 Applications of Soybean in Food and Industrial Sector

Alternative animal proteins, also known as meat analogues and substitutions, have emerged as a result of the search for wholesome and sustainable plant‐based food options. Soy is one such protein that is both adaptable and popular. Due to this growing need, a wide variety of soy‐based goods have been developed, such as deli meats, sausages, and burgers, which have become quite popular in the market for plant‐based protein substitutes (Messina et al. 2022). In order to create meat analogues and substitutes that mimic the flavor, texture, and appearance of real meat while offering a sustainable protein supply, soy is a key component. The environmental sustainability of using soy as a beef protein substitute is one of its main benefits. Conventional animal farming contributes to deforestation and greenhouse gas emissions, requires a large amount of land, and uses a lot of water and feed resources.

“Okara” is the term for the soy residue that is produced as a result of processing soy‐derived products. An insoluble byproduct of processing soymilk, tofu, and soy nuts is called okara. Because okara includes lactose (a prebiotic source) and probiotics, fermentation improves its functional qualities and may increase its usefulness (Voss et al. 2021). Eicosapentaenoic acid (EPA) and fucoxanthin are two nutraceuticals made from fermented okara (Kim et al. 2023).

The most traded, processed, and globally distributed agricultural commodity is soybean. The market for soybean derivatives can be split into four categories: (1) by type, which includes (i) soybeans, (ii) soymeal (soy milk and soy protein concentrate), and (iii) soy oil (soy lecithin); (2) by application, which includes (i) feed, (ii) food, and (iii) other industries like biodiesel, soy‐based wood adhesives, soy ink, soy crayons, soy‐based lubricants, and many more; (3) by lecithin processing, which includes (i) water, (ii) acid, and (iii) enzyme; and (4) by region, which includes North and South America, Europe, China, Japan, Southeast Asia, South Asia, and South Asia (India) (S. P. Tiwari 2017). The industrial processing of soybeans can be summed up in two steps: (1) producing crude oil from soybean residue and (2) refining crude oil to produce various products, such as hydrogenated fat, margarine, and refined oils. Since biodiesel is seen as a renewable and sustainable energy source, it is currently a significant biofuel of international importance (de Mello et al. 2017). Fuel generation has the ability to close the carbon cycle because refined soy oil is the most commonly used raw material. Furthermore, although biodiesel has higher NOx emissions than fossil fuel, it produces fewer pollutants overall, primarily CO_2_, CO, and SO_2_ (Ang et al. 2014).

### 13.4 Emerging Health Benefits

The bioactive ingredients in soybeans, particularly isoflavones, improve immunity and digestion while preventing cancer, osteoporosis, CVD, and microbial infections. Compared to other polyphenols, isoflavones (genistein and daidzein), aglycones, and glycosides exhibit potent antioxidant qualities. Interestingly, these antioxidants stop the oxidation of low‐density lipoproteins and damage to DNA. It is evident from the explanation above that okara is a major source of bioactive ingredients (Kamble and Rani 2020).

Soybean and its bioactive components have several health benefits. According to studies, isoflavones improve existing neural function, promote neuronal regeneration, and guard against neuronal cell death. Therefore, because of their purportedly advantageous properties, such as genistein's capacity to block the apoptotic signaling cascade in neurons, interest in consuming fermented soybean meals high in isoflavones is increasing (Dias et al. 2012).

Studies on the effects of fermented soy products on the control of SARS‐CoV‐2 are also carried out. A study with natto is being conducted to examine the antiviral properties of this food against SARS‐CoV‐2. The findings demonstrated that natto extract completely prevented the cells from becoming infected with the severe acute respiratory syndrome coronavirus 2 (SARS‐CoV‐2). The British form of the spike protein (receptor binding domain; RBD) of SARS‐CoV‐2 was proteolytically broken down by the protease activities of natto extract, which prevented viral infections in cells (Oba et al. 2021).

## 14 Conclusions

Offering bioactive substances like isoflavones, saponins, and premium proteins with shown health advantages, including cardioprotective, anti‐inflammatory, and anticancer properties, soybeans are a nutrient‐dense and functionally diverse crop. Recent developments in innovative extraction methods, including enzymatic hydrolysis, supercritical fluid extraction, microwave‐assisted extraction, and ultrasound‐assisted extraction, have shown great promise in improving the yield, purity, and bioactivity of compounds derived from soybeans while lowering processing time, energy costs, and environmental effects. In addition to increasing the functional and nutraceutical value of soybean products, these creative methods pave the way for the creation of medicinal agents and tailored functional meals.

## Author Contributions

**Muhammad Rashid:** writing – original draft (equal). **Iqra Khalid:** resources (equal). **Gaurav Sanghvi:** visualization (equal). **Amar Shankar:** validation (equal). **Farhan Saeed:** validation (equal). **Muhammad Afzaal:** validation (equal). software (equal). **Mayank Kundlas:** formal analysis (equal). **Mehwish:** data curation (equal). **Catherine Tamale Ndagire:** software (equal).

## Consent

All the co‐authors are willing to participate in this manuscript and also for the publication of this manuscript.

## Conflicts of Interest

The authors declare no conflicts of interest.

## Data Availability

The datasets generated, used and/or analyzed during the current study are available from the corresponding author on reasonable request.
